# Synthesis and crystal structure of *trans*-di­aqua(1,4,8,11-tetra­aza­undeca­ne)copper(II) isophthalate monohydrate

**DOI:** 10.1107/S2056989022007538

**Published:** 2022-07-29

**Authors:** Liudmyla V. Tsymbal, Vladimir B. Arion, Yaroslaw D. Lampeka

**Affiliations:** a L. V. Pisarzhevskii Institute of Physical Chemistry of the National Academy of Sciences of Ukraine, Prospekt Nauki 31, 03028 Kiev, Ukraine; b Institute of Inorganic Chemistry of the University of Vienna, Wahringer Str., 42, 1090 Vienna, Austria; University of Aberdeen, Scotland

**Keywords:** crystal structure, tetra­amine ligand, copper, isophthalic acid, hydrogen bonds

## Abstract

In the title hydrated mol­ecular salt, [Cu(C_7_H_20_N_4_)(H_2_O)_2_](C_8_H_4_O_4_)·H_2_O, the metal ion is coordinated by the two primary and two secondary N atoms of the amine ligand and the mutually *trans* O atoms of the water mol­ecules in a tetra­gonally distorted octa­hedral geometry. The average equatorial Cu—N bond lengths (2.013 and 2.026 Å for Cu—N_prim_ and Cu—N_sec_, respectively) are substanti­ally shorter than the average axial Cu—O bond length (2.518 Å). The tetra­amine ligand adopts its energetically favored conformation with its five- and six-membered chelate rings in *gauche* and chair conformations, respectively. In the crystal, the N—H donor groups of the tetra­amine, the acceptor carboxyl­ate groups of the isophthalate dianion and both the coordinated water mol­ecules and the water mol­ecule of crystallization are involved in numerous N—H⋯O and O—H⋯O hydrogen bonds, resulting in the formation of electroneutral layers oriented parallel to the *ac* plane.

## Abstract 2

The complex cation of the title compound contains a tetra­gonally distorted *trans*-CuN_4_O_2_ octa­hedron. In the crystal, the components are linked by numerous N—H⋯O and O—H⋯O hydrogen bonds, forming electroneutral sheets oriented parallel to the *ac* plane, which are further consolidated into bilayers due to hydrogen-bonding with the participation of the water mol­ecule of crystallization.

## 1. Chemical context

The copper(II) and nickel(II) complexes of tetra­dentate aza­macrocyclic ligands, in particular, cyclam and its structural analogues (cyclam = 1,4,8,11-tetra­aza­cyclo­tetra­decane, C_10_H_24_N_4_), are widely used for the construction of metal–organic frameworks (MOFs) based on oligo­carboxyl­ate linkers, which possess many promising applications (Lampeka & Tsymbal, 2004 ▸; Suh & Moon, 2007 ▸; Suh *et al.*, 2012 ▸; Stackhouse & Ma, 2018 ▸; Lee & Moon, 2018 ▸). At the same time, open-chain aliphatic tetra­amines like *L* (*L* = 1,4,8,11-tetra­aza­undecane, C_7_H_20_N_4_), which is the closest structural and electronic analogue of cyclam, are practically unexploited in this respect and only one work dealing with the crystal structures of MOFs formed by the [Ni(*L*)]^2+^ cation with tris­(4-carboxyl­atobenz­yl)amine has been reported to date (Jiang *et al.*, 2012 ▸). Besides, the [*M*(*L*)] synthons (*M* = Cu^II^, Ni^II^) are convenient precursors for the one-pot template preparation of corresponding metal complexes of 14-membered aza­cyclam macrocycles (aza­cyclam = 1,4,8,11,13-penta­aza­cyclo­tetra­deca­ne) (Rosokha *et al.*, 1993 ▸; Gerbeleu *et al.*, 1999 ▸) and some complexes of this type functionalized at the N^13^ position of the macrocyclic backbone have been structurally characterized by our group (Andriichuk *et al.*, 2019 ▸; Tsymbal *et al.*, 2010 ▸, 2021 ▸). Herein, we report the syntheses and crystal structure of the product of the reaction of CuCl_2_, *L* and the isophthalate anion (ip^2−^) as its sodium salt, namely, *trans*-di­aqua­(1,4,8,11-tetra­aza­undecane-*κ* ^4^ *N* ^1^,*N* ^4^,*N* ^8^,*N* ^11^)-copper(II) isophthalate monohydrate, [Cu(*L*)(H_2_O)_2_](ip)·H_2_O, **I**. 
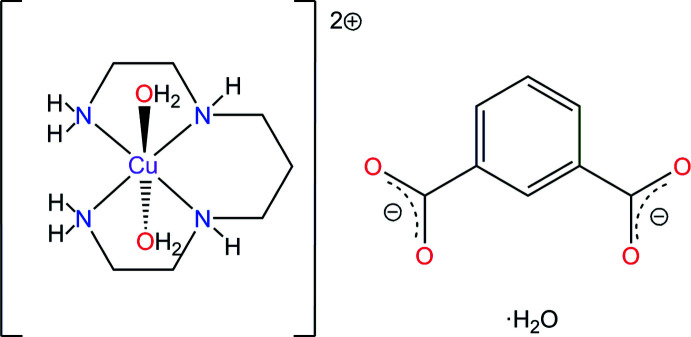


## 2. Structural commentary

**Figure 1:**
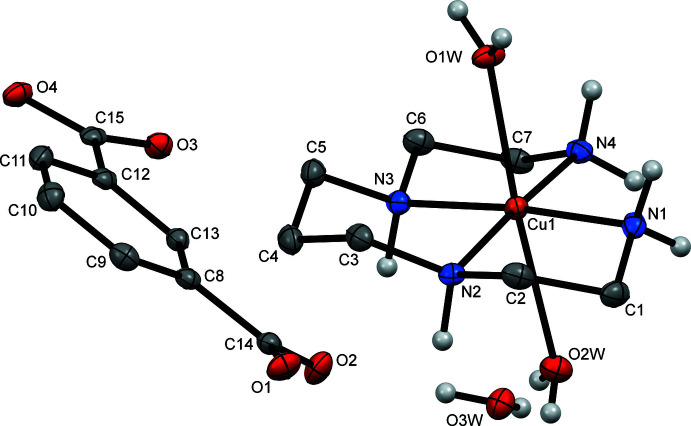
View of the asymmetric unit of **I**, showing the atom-labelling scheme, with displacement ellipsoids drawn at the 40% probability level. H atoms attached to carbon atoms have been omitted for clarity.

The asymmetric unit of the title hydrated mol­ecular salt **I** consists of a complex di-cation [Cu(*L*)(H_2_O)_2_]^2+^, a non-coord­inated isophthalate di-anion ip^2–^ and one water mol­ecule of crystallization (Fig. 1 ▸). The Cu^II^ ion is coordinated in the equatorial plane by the two primary and two secondary N atoms of the amine ligand in a nearly square-planar fashion (the deviations of the N atoms from the mean N_4_ plane are ±0.006 Å), and by the two O atoms from the water mol­ecules in the axial positions.

The average equatorial Cu—N_*prim*_ bond length for N1 and N4 (2.013 Å) is slightly shorter than Cu—N_*sec*_ one for N2 and N3 (2.025 Å), probably reflecting the stronger donating ability of the N atoms of primary *versus* secondary amine groups (Table 1 ▸). The average axial Cu—O bond length (2.518 Å) is substanti­ally longer than the equatorial Cu—N bonds, which is likely due to a large Jahn–Teller distortion inherent in metal ions with a *d* ^9^ electronic configuration. It is noteworthy that the Cu—O distances in **I** differ considerably (Table 1 ▸) and the Cu^II^ ion is displaced from the mean N_4_ plane of the ligand by 0.082 Å towards the O1*W* water mol­ecule.

The ligand *L* in **I** adopts its energetically favored conformation with the five-membered chelate rings in *gauche* [average bite angle 85.74°] and six-membered chelate ring in *chair* conformations, which resemble the *trans*-III conformation usually observed in cyclam complexes (Barefield *et al.*, 1986 ▸; Bosnich *et al.*, 1965 ▸). The pseudo ‘bite’ angle formed by the primary amine donors N1—Cu1—N4 is slightly larger than that for N2—Cu1—N3 (Table 1 ▸).

The isophthalate di-anion in the title compound counterbalances the charge of the complex cation. The mean planes of the pendant carboxyl­ate groups are slightly tilted relative to the mean plane of the aromatic ring [average angle = 9.8°]. The C—O bond lengths in the carboxyl­ate groups are nearly equal (Table 1 ▸), thus indicating essentially complete electron delocalization.

## 3. Supra­molecular features

**Figure 2:**
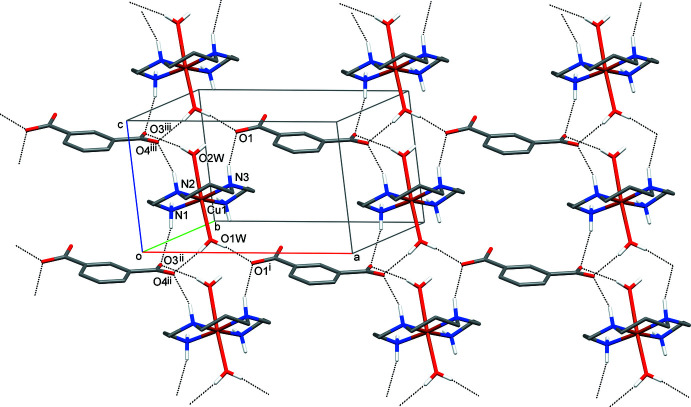
The hydrogen-bonded (dashed lines) sheets in **I**. C-bound H atoms and water mol­ecule of crystallization have been omitted.

**Figure 3:**
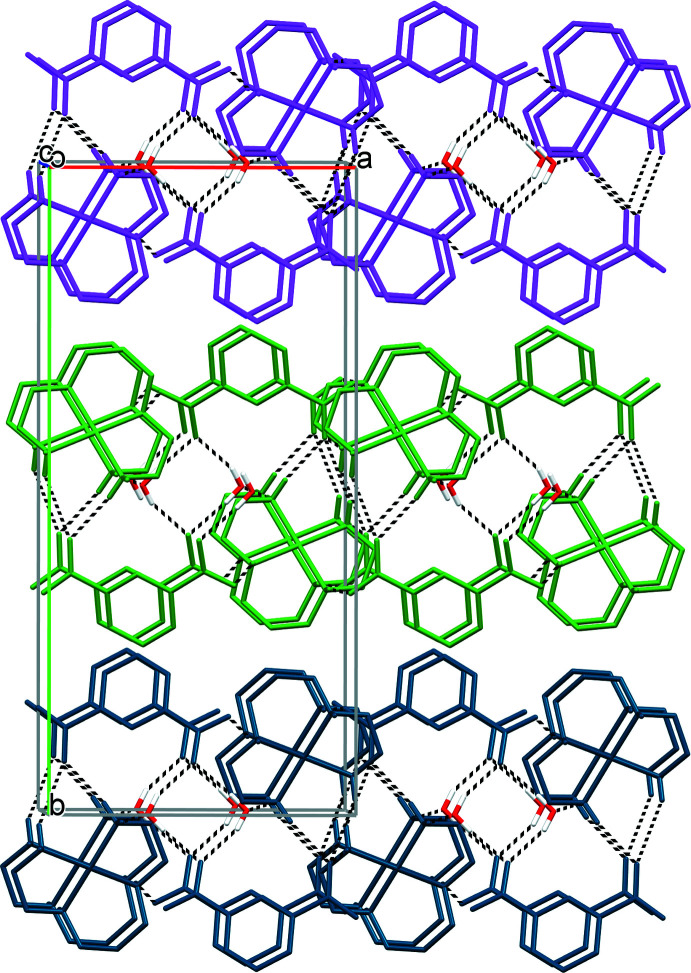
Side view of the bilayers in **I** along the *c* axis. C-bound H atoms and coordinated water mol­ecules have been omitted, hydrogen bonds are shown as dashed lines.

In the crystal of **I**, the complex cation [Cu(*L*)(H_2_O)_2_]^2+^, isophthalate anion ip^2–^ and both coordinated water mol­ecules and water mol­ecule of crystallization are linked by numerous hydrogen bonds (Table 2 ▸), resulting in its distinct lamellar structure. In particular, hydrogen-bonding inter­actions between the N1, N2 and N3 amine groups and O1*W* and O2*W* water mol­ecules as the donors and carboxyl­ate atoms O1, O3 and O4 as the acceptors result in the formation of electroneutral sheets (Fig. 2 ▸). Additionally, due to hydrogen bonds N4—H4*B*⋯O3 (−*x* + 1, −*y* + 1, −*z* + 1) and N1—H1*A*⋯O2*W* (−*x*, −*y* + 1, −*z* + 1) and four bonds formed by the water mol­ecule O3*W* these sheets double into bilayers oriented parallel to the *ac* plane (Fig. 3 ▸). It is noteworthy that all the polar groups in **I** are saturated from the point of view of the number of possible hydrogen bonds, which equal to 2, 1, 2, 4 and 2 for the primary, secondary amine groups, coordinated water mol­ecule, water mol­ecule of crystallization and carboxyl­ate O atoms, respectively.

There are no hydrogen-bonding contacts between the layers in **I** (Fig. 3 ▸). The three-dimensional coherence of the crystal is provided by van der Waals inter­actions between the methine and methyl­ene fragments of the constituents.

## 4. Database survey

A search of the Cambridge Structural Database (CSD, version 5.43, last update March 2022; Groom *et al.*, 2016 ▸) gave nine hits related to the compounds formed by the [Cu(*L*)]^2+^ core. Among them, the *trans*-CuN_4_O_2_ chromophores are characteristic of three complexes [CSD refcodes DAFYOA (Heeg *et al.*, 2010 ▸), FICDEA (Lawrance *et al.*, 1987 ▸) and TECCUA (Fawcett *et al.*, 1980 ▸)] all of which contain coordinated perchlorate anions. Thus, the present work is the first structural characterization of a Cu^II^ di­aqua complex of this open-chain tetra­amine.

In general, conformations of the amine ligand and geometrical parameters of coordination polyhedra in both types of cations are similar, even though the axial Cu—O bond lengths in the perchlorate complexes are longer. This can be explained by poorer donating ability of this anion as compared to aqua ligand. As in **I**, the Cu—O distances in previously mentioned compounds are non-equivalent even though the differences between them are smaller than in **I** and do not exceed 0.14 Å.

## 5. Synthesis and crystallization

All chemicals and solvents used in this work were purchased from Sigma–Aldrich and used without further purification. The title compound **I** was prepared as follows. A solution of Na_2_ip (105 mg, 0.5 mmol) in water (5 ml) was added to a solution of CuCl_2_·2H_2_O (85 mg, 0.5 mmol) and *L* (80 mg (0.5 mmol) in water (5 ml). The blue precipitate, which formed in several days, was filtered off, washed with methanol (2 ml) and diethyl ether and dried in air. Yield: 106 mg (48%). Analysis calculated for C_15_H_30_CuN_4_O_7_: C 40.76, H 6.84, N 12.67%. Found: C 40.56, H 6.96, N 12.42%. Single crystals of **I** of X-ray diffraction quality were selected from the sample resulting from the synthesis.

## 6. Refinement

Crystal data, data collection and structure refinement details are summarized in Table 3 ▸. H atoms in **I** were placed in geometrically idealized positions and constrained to ride on their parent atoms, with C—H distances of 0.95 (ring H atoms) or 0.99 Å (aliphatic H atoms), N—H distances of 0.97 (primary amine groups) or 0.98 Å (secondary amine groups) with *U*_iso_(H) values of 1.2*U*_eq_ of the parent atoms. Water H atoms were positioned geometrically (O—H distances of 0.87 Å) and refined as riding with *U*_iso_(H) = 1.5*U*_eq_(O).

## Supplementary Material

Crystal structure: contains datablock(s) I. DOI: 10.1107/S2056989022007538/hb8031sup1.cif

Structure factors: contains datablock(s) I. DOI: 10.1107/S2056989022007538/hb8031Isup2.hkl

CCDC reference: 2190232

Additional supporting information:  crystallographic information; 3D view; checkCIF report

## Figures and Tables

**Table 1:** Selected geometric parameters (Å, °)

Cu1—N1	2.0203 (18)	Cu1—O2*W*	2.6562 (16)
Cu1—N2	2.0218 (18)	C14—O1	1.256 (3)
Cu1—N3	2.0279 (18)	C14—O2	1.261 (3)
Cu1—N4	2.0064 (19)	C15—O3	1.258 (3)
Cu1—O1*W*	2.3800 (16)	C15—O4	1.271 (3)
			
N1—Cu1—N2	85.64 (8)	N4—Cu1—N1	95.19 (8)
N2—Cu1—N3	92.97 (7)	N4—Cu1—N3	85.83 (8)

**Table 2:** Hydrogen-bond geometry (Å, °)

*D*—H⋯*A*	*D*—H	H⋯*A*	*D*⋯*A*	*D*—H⋯*A*
N1—H1*A*⋯O2*W* ^i^	0.97	2.30	3.143 (2)	145
N1—H1*B*⋯O3^ii^	0.97	2.07	3.007 (2)	161
N2—H2⋯O4^iii^	0.98	1.95	2.907 (2)	163
N3—H3⋯O1	0.98	2.19	3.063 (3)	148
N4—H4*A*⋯O3*W* ^iv^	0.97	2.10	3.042 (2)	163
N4—H4*B*⋯O3^v^	0.97	2.17	3.054 (2)	151
O1*W*—H1*WA*⋯O1^iv^	0.87	1.89	2.747 (2)	169
O1*W*—H1*WB*⋯O4^ii^	0.87	1.89	2.760 (2)	174
O2*W*—H2*WA*⋯O3^iii^	0.87	2.09	2.930 (2)	161
O2*W*—H2*WB*⋯O3*W*	0.87	2.00	2.872 (2)	175
O3*W*—H3*WA*⋯O2^vi^	0.87	2.00	2.823 (2)	157
O3*W*—H3*WB*⋯O2	0.87	1.85	2.712 (2)	174

Symmetry codes: (i) 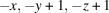 ; (ii) 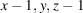 ; (iii) 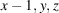 ; (iv) 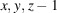 ; (v) 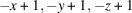 ; (vi) 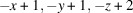 .

**Table 3:** Experimental details

Crystal data	
Chemical formula	[Cu(C_7_H_20_N_4_)(H_2_O)_2_](C_8_H_4_O_4_)·H_2_O
*M*_r_	441.97
Crystal system, space group	Monoclinic, *P*2_1_/*c*
Temperature (K)	100
*a*, *b*, *c* (Å)	11.4727 (8), 24.1694 (18), 7.1591 (5)
β (°)	96.679 (4)
*V* (Å^3^)	1971.7 (2)
*Z*	4
Radiation type	Mo *K*α
μ (mm^−1^)	1.15
Crystal size (mm)	0.15 × 0.15 × 0.06

Data collection	
Diffractometer	Bruker APEXII CCD
Absorption correction	Multi-scan (*SADABS*; Krause *et al.*, 2015 ▸)
*T*_min_, *T*_max_	0.846, 0.934
No. of measured, independent and observed [*I* > 2σ(*I*)] reflections	53784, 3698, 3232
*R*_int_	0.050
(sin θ/λ)_max_ (Å^−1^)	0.608

Refinement	
*R*[*F* ^2^ > 2σ(*F* ^2^)], *wR*(*F* ^2^), *S*	0.032, 0.079, 1.11
No. of reflections	3698
No. of parameters	248
No. of restraints	11
H-atom treatment	H atoms treated by a mixture of independent and constrained refinement
Δρ_max_, Δρ_min_ (e Å^−3^)	0.50, −0.32

Computer programs: *APEX2* and *SAINT* (Bruker, 2012 ▸), *SHELXT2018/2* (Sheldrick, 2015*a* ▸), *SHELXL2018/3* (Sheldrick, 2015*b* ▸), *Mercury* (Macrae *et al.*, 2020 ▸) and *publCIF* (Westrip, 2010 ▸).

## supplementary crystallographic information

### Crystal data

**Table:**

[Cu(C_7_H_20_N_4_)(H_2_O)_2_](C_8_H_4_O_4_)·H_2_O	*F*(000) = 932
*M**_r_* = 441.97	*D*_x_ = 1.489 Mg m^−^^3^
Monoclinic, *P*2_1_/*c*	Mo *K*α radiation, λ = 0.71073 Å
*a* = 11.4727 (8) Å	Cell parameters from 2350 reflections
*b* = 24.1694 (18) Å	θ = 2.0–25.0°
*c* = 7.1591 (5) Å	µ = 1.15 mm^−^^1^
β = 96.679 (4)°	*T* = 100 K
*V* = 1971.7 (2) Å^3^	Prism, light blue
*Z* = 4	0.15 × 0.15 × 0.06 mm

### Data collection

**Table:**

Bruker APEXII CCD diffractometer	3232 reflections with *I* > 2σ(*I*)
φ and ω scans	*R*_int_ = 0.050
Absorption correction: multi-scan (SADABS; Krause *et al.*, 2015)	θ_max_ = 25.6°, θ_min_ = 2.0°
*T*_min_ = 0.846, *T*_max_ = 0.934	*h* = −13→13
53784 measured reflections	*k* = −29→29
3698 independent reflections	*l* = −8→8

### Refinement

**Table:**

Refinement on *F*^2^	Primary atom site location: dual
Least-squares matrix: full	Hydrogen site location: mixed
*R*[*F*^2^ > 2σ(*F*^2^)] = 0.032	H atoms treated by a mixture of independent and constrained refinement
*wR*(*F*^2^) = 0.079	*w* = 1/[σ^2^(*F*_o_^2^) + (0.0332*P*)^2^ + 1.9773*P*] where *P* = (*F*_o_^2^ + 2*F*_c_^2^)/3
*S* = 1.11	(Δ/σ)_max_ = 0.001
3698 reflections	Δρ_max_ = 0.50 e Å^−^^3^
248 parameters	Δρ_min_ = −0.32 e Å^−^^3^
11 restraints	

### Special details

**Table:**

Geometry. All esds (except the esd in the dihedral angle between two l.s. planes) are estimated using the full covariance matrix. The cell esds are taken into account individually in the estimation of esds in distances, angles and torsion angles; correlations between esds in cell parameters are only used when they are defined by crystal symmetry. An approximate (isotropic) treatment of cell esds is used for estimating esds involving l.s. planes.

### Fractional atomic coordinates and isotropic or equivalent isotropic displacement parameters (Å^2^)

**Table:**

	*x*	*y*	*z*	*U*_iso_*/*U*_eq_	
Cu1	0.16222 (2)	0.41162 (2)	0.30691 (4)	0.01549 (9)	
O1W	0.19539 (13)	0.37755 (7)	0.0057 (2)	0.0222 (4)	
H1WB	0.140042	0.364422	−0.075635	0.033*	
H1WA	0.262802	0.372482	−0.036104	0.033*	
N1	0.00154 (16)	0.44151 (8)	0.2133 (3)	0.0187 (4)	
H1B	−0.009406	0.441387	0.076913	0.022*	
H1A	−0.008806	0.479637	0.250343	0.022*	
N2	0.07640 (16)	0.34306 (8)	0.3776 (3)	0.0162 (4)	
H2	0.070058	0.343234	0.512940	0.019*	
N3	0.31993 (16)	0.38284 (8)	0.4245 (3)	0.0173 (4)	
H3	0.320619	0.384199	0.561434	0.021*	
N4	0.24838 (16)	0.48139 (8)	0.2576 (3)	0.0185 (4)	
H4B	0.207629	0.515358	0.281406	0.022*	
H4A	0.264879	0.480508	0.127836	0.022*	
C1	−0.0875 (2)	0.40536 (10)	0.2874 (3)	0.0225 (5)	
H1C	−0.164157	0.409352	0.209563	0.027*	
H1D	−0.097246	0.415929	0.418270	0.027*	
C2	−0.04516 (19)	0.34645 (10)	0.2813 (3)	0.0210 (5)	
H2A	−0.097361	0.321866	0.344534	0.025*	
H2B	−0.046743	0.334230	0.148997	0.025*	
C3	0.1331 (2)	0.28961 (10)	0.3430 (3)	0.0208 (5)	
H3A	0.138014	0.285553	0.206536	0.025*	
H3B	0.084020	0.259027	0.382426	0.025*	
C4	0.2563 (2)	0.28505 (10)	0.4492 (3)	0.0223 (5)	
H4C	0.251528	0.292366	0.584128	0.027*	
H4D	0.284504	0.246613	0.438010	0.027*	
C5	0.3461 (2)	0.32440 (10)	0.3804 (3)	0.0213 (5)	
H5A	0.425481	0.314656	0.440994	0.026*	
H5B	0.345745	0.320183	0.242808	0.026*	
C6	0.4108 (2)	0.42145 (10)	0.3695 (3)	0.0214 (5)	
H6A	0.429531	0.412384	0.241485	0.026*	
H6B	0.483487	0.417883	0.457819	0.026*	
C7	0.3643 (2)	0.48003 (10)	0.3738 (3)	0.0216 (5)	
H7A	0.355909	0.491074	0.504759	0.026*	
H7B	0.419204	0.506014	0.322469	0.026*	
O1	0.39258 (13)	0.35317 (7)	0.8377 (2)	0.0242 (4)	
O2	0.50998 (14)	0.42314 (7)	0.9402 (2)	0.0241 (4)	
O3	0.92569 (13)	0.42289 (7)	0.8018 (2)	0.0199 (4)	
O4	1.00945 (13)	0.34100 (7)	0.7569 (2)	0.0215 (4)	
C8	0.59710 (19)	0.34397 (9)	0.8164 (3)	0.0159 (5)	
C9	0.5866 (2)	0.28909 (10)	0.7585 (3)	0.0197 (5)	
H9	0.512981	0.270876	0.754070	0.024*	
C10	0.6837 (2)	0.26072 (10)	0.7071 (3)	0.0220 (5)	
H10	0.676259	0.223180	0.668228	0.026*	
C11	0.7912 (2)	0.28737 (10)	0.7129 (3)	0.0202 (5)	
H11	0.857145	0.267874	0.677579	0.024*	
C12	0.80347 (19)	0.34233 (9)	0.7697 (3)	0.0167 (5)	
C13	0.70586 (19)	0.37043 (9)	0.8219 (3)	0.0158 (4)	
H13	0.713443	0.407907	0.861456	0.019*	
C14	0.49156 (19)	0.37561 (9)	0.8704 (3)	0.0171 (5)	
C15	0.92127 (19)	0.37139 (10)	0.7764 (3)	0.0172 (5)	
O2W	0.13469 (14)	0.45839 (7)	0.6347 (2)	0.0227 (4)	
H2WA	0.083740	0.442503	0.698016	0.039 (9)*	
H2WB	0.194230	0.469203	0.712486	0.040 (9)*	
O3W	0.33455 (14)	0.49839 (7)	0.8756 (2)	0.0224 (4)	
H3WA	0.369284	0.528915	0.915066	0.029 (7)*	
H3WB	0.387514	0.472565	0.893226	0.044 (9)*	

### Atomic displacement parameters (Å^2^)

**Table:**

	*U*^11^	*U*^22^	*U*^33^	*U*^12^	*U*^13^	*U*^23^
Cu1	0.01437 (15)	0.01537 (15)	0.01669 (15)	−0.00035 (10)	0.00163 (10)	0.00072 (11)
O1W	0.0143 (8)	0.0350 (10)	0.0176 (8)	−0.0001 (7)	0.0028 (6)	−0.0051 (7)
N1	0.0202 (10)	0.0174 (10)	0.0180 (10)	0.0036 (8)	−0.0001 (8)	−0.0015 (8)
N2	0.0166 (9)	0.0184 (10)	0.0138 (9)	−0.0012 (7)	0.0025 (7)	−0.0009 (7)
N3	0.0173 (9)	0.0188 (10)	0.0158 (10)	0.0003 (8)	0.0025 (7)	0.0005 (8)
N4	0.0231 (10)	0.0155 (10)	0.0173 (10)	−0.0019 (8)	0.0039 (8)	−0.0003 (8)
C1	0.0154 (11)	0.0325 (14)	0.0197 (12)	0.0018 (10)	0.0024 (9)	0.0009 (10)
C2	0.0166 (11)	0.0283 (13)	0.0177 (12)	−0.0059 (9)	0.0005 (9)	0.0007 (10)
C3	0.0266 (12)	0.0168 (12)	0.0188 (12)	−0.0022 (9)	0.0024 (9)	−0.0011 (9)
C4	0.0277 (13)	0.0170 (12)	0.0217 (13)	0.0032 (10)	0.0010 (10)	0.0013 (9)
C5	0.0200 (12)	0.0231 (13)	0.0204 (12)	0.0055 (9)	0.0002 (9)	0.0006 (10)
C6	0.0160 (11)	0.0284 (14)	0.0196 (12)	−0.0039 (9)	0.0012 (9)	0.0019 (10)
C7	0.0220 (12)	0.0247 (13)	0.0180 (12)	−0.0079 (10)	0.0019 (9)	0.0005 (10)
O1	0.0139 (8)	0.0326 (10)	0.0264 (9)	−0.0032 (7)	0.0042 (7)	−0.0062 (8)
O2	0.0180 (8)	0.0205 (9)	0.0344 (10)	0.0007 (7)	0.0064 (7)	−0.0047 (7)
O3	0.0163 (8)	0.0221 (9)	0.0213 (9)	−0.0015 (6)	0.0027 (6)	0.0006 (7)
O4	0.0141 (8)	0.0318 (10)	0.0189 (8)	0.0030 (7)	0.0029 (6)	−0.0025 (7)
C8	0.0160 (11)	0.0201 (12)	0.0115 (11)	−0.0006 (9)	0.0004 (8)	0.0027 (9)
C9	0.0196 (12)	0.0212 (12)	0.0177 (12)	−0.0045 (9)	−0.0005 (9)	0.0014 (9)
C10	0.0253 (12)	0.0192 (12)	0.0210 (12)	0.0013 (10)	−0.0002 (9)	−0.0023 (10)
C11	0.0202 (11)	0.0234 (13)	0.0170 (12)	0.0062 (9)	0.0021 (9)	−0.0006 (9)
C12	0.0153 (11)	0.0240 (12)	0.0103 (10)	0.0017 (9)	0.0000 (8)	0.0026 (9)
C13	0.0189 (11)	0.0151 (11)	0.0132 (11)	0.0006 (9)	0.0013 (8)	0.0009 (9)
C14	0.0180 (11)	0.0201 (12)	0.0134 (11)	0.0002 (9)	0.0031 (8)	0.0024 (9)
C15	0.0162 (11)	0.0267 (13)	0.0085 (10)	0.0019 (9)	0.0010 (8)	0.0007 (9)
O2W	0.0220 (9)	0.0260 (9)	0.0201 (9)	−0.0036 (7)	0.0021 (7)	0.0005 (7)
O3W	0.0202 (8)	0.0216 (9)	0.0249 (9)	0.0006 (7)	0.0005 (7)	−0.0031 (7)

### Geometric parameters (Å, º)

**Table:**

Cu1—N1	2.0203 (18)	C4—H4D	0.9900
Cu1—N2	2.0218 (18)	C4—C5	1.526 (3)
Cu1—N3	2.0279 (18)	C5—H5A	0.9900
Cu1—N4	2.0064 (19)	C5—H5B	0.9900
Cu1—O1W	2.3800 (16)	C6—H6A	0.9900
Cu1—O2W	2.6562 (16)	C6—H6B	0.9900
O1W—H1WB	0.8700	C6—C7	1.514 (3)
O1W—H1WA	0.8698	C7—H7A	0.9900
N1—H1B	0.9699	C7—H7B	0.9900
N1—H1A	0.9701	C14—O1	1.256 (3)
N1—C1	1.488 (3)	C14—O2	1.261 (3)
N2—H2	0.9798	C15—O3	1.258 (3)
N2—C2	1.485 (3)	C15—O4	1.271 (3)
N2—C3	1.480 (3)	C8—C9	1.390 (3)
N3—H3	0.9799	C8—C13	1.399 (3)
N3—C5	1.486 (3)	C8—C14	1.520 (3)
N3—C6	1.486 (3)	C9—H9	0.9500
N4—H4B	0.9696	C9—C10	1.393 (3)
N4—H4A	0.9699	C10—H10	0.9500
N4—C7	1.485 (3)	C10—C11	1.388 (3)
C1—H1C	0.9900	C11—H11	0.9500
C1—H1D	0.9900	C11—C12	1.392 (3)
C1—C2	1.506 (3)	C12—C13	1.397 (3)
C2—H2A	0.9900	C12—C15	1.519 (3)
C2—H2B	0.9900	C13—H13	0.9500
C3—H3A	0.9900	O2W—H2WA	0.8698
C3—H3B	0.9900	O2W—H2WB	0.8699
C3—C4	1.529 (3)	O3W—H3WA	0.8699
C4—H4C	0.9900	O3W—H3WB	0.8699
			
O1W—Cu1—O2W	174.64 (6)	N2—C3—C4	112.24 (19)
N1—Cu1—O1W	93.38 (7)	H3A—C3—H3B	107.9
N1—Cu1—N2	85.64 (8)	C4—C3—H3A	109.2
N1—Cu1—N3	174.88 (8)	C4—C3—H3B	109.2
N1—Cu1—O2W	86.75 (6)	C3—C4—H4C	108.6
N2—Cu1—O1W	94.42 (7)	C3—C4—H4D	108.6
N2—Cu1—N3	92.97 (7)	H4C—C4—H4D	107.6
N2—Cu1—O2W	90.93 (6)	C5—C4—C3	114.52 (19)
N3—Cu1—O1W	91.63 (7)	C5—C4—H4C	108.6
N3—Cu1—O2W	88.35 (6)	C5—C4—H4D	108.6
N4—Cu1—O1W	89.86 (7)	N3—C5—C4	111.29 (19)
N4—Cu1—N1	95.19 (8)	N3—C5—H5A	109.4
N4—Cu1—N2	175.59 (7)	N3—C5—H5B	109.4
N4—Cu1—N3	85.83 (8)	C4—C5—H5A	109.4
N4—Cu1—O2W	84.80 (6)	C4—C5—H5B	109.4
Cu1—O1W—H1WB	123.5	H5A—C5—H5B	108.0
Cu1—O1W—H1WA	127.1	N3—C6—H6A	109.9
H1WB—O1W—H1WA	109.1	N3—C6—H6B	109.9
Cu1—N1—H1B	109.8	N3—C6—C7	108.80 (18)
Cu1—N1—H1A	112.6	H6A—C6—H6B	108.3
H1B—N1—H1A	105.8	C7—C6—H6A	109.9
C1—N1—Cu1	108.02 (14)	C7—C6—H6B	109.9
C1—N1—H1B	110.1	N4—C7—C6	107.68 (18)
C1—N1—H1A	110.5	N4—C7—H7A	110.2
Cu1—N2—H2	109.8	N4—C7—H7B	110.2
C2—N2—Cu1	107.27 (14)	C6—C7—H7A	110.2
C2—N2—H2	106.6	C6—C7—H7B	110.2
C3—N2—Cu1	115.92 (14)	H7A—C7—H7B	108.5
C3—N2—H2	104.7	C9—C8—C13	119.4 (2)
C3—N2—C2	112.19 (18)	C9—C8—C14	120.8 (2)
Cu1—N3—H3	107.9	C13—C8—C14	119.8 (2)
C5—N3—Cu1	115.52 (14)	C8—C9—H9	119.9
C5—N3—H3	105.4	C8—C9—C10	120.3 (2)
C5—N3—C6	112.04 (18)	C10—C9—H9	119.9
C6—N3—Cu1	107.10 (14)	C9—C10—H10	120.0
C6—N3—H3	108.6	C11—C10—C9	119.9 (2)
Cu1—N4—H4B	115.0	C11—C10—H10	120.0
Cu1—N4—H4A	107.8	C10—C11—H11	119.6
H4B—N4—H4A	109.8	C10—C11—C12	120.7 (2)
C7—N4—Cu1	108.08 (14)	C12—C11—H11	119.6
C7—N4—H4B	109.8	C11—C12—C13	119.0 (2)
C7—N4—H4A	105.9	C11—C12—C15	120.7 (2)
N1—C1—H1C	110.1	C13—C12—C15	120.3 (2)
N1—C1—H1D	110.1	C8—C13—H13	119.7
N1—C1—C2	107.93 (18)	C12—C13—C8	120.7 (2)
H1C—C1—H1D	108.4	C12—C13—H13	119.7
C2—C1—H1C	110.1	O1—C14—O2	125.0 (2)
C2—C1—H1D	110.1	O1—C14—C8	117.7 (2)
N2—C2—C1	109.08 (18)	O2—C14—C8	117.24 (19)
N2—C2—H2A	109.9	O3—C15—O4	124.6 (2)
N2—C2—H2B	109.9	O3—C15—C12	118.88 (19)
C1—C2—H2A	109.9	O4—C15—C12	116.5 (2)
C1—C2—H2B	109.9	Cu1—O2W—H2WA	115.4
H2A—C2—H2B	108.3	Cu1—O2W—H2WB	121.9
N2—C3—H3A	109.2	H2WA—O2W—H2WB	108.9
N2—C3—H3B	109.2	H3WA—O3W—H3WB	106.0
			
Cu1—N1—C1—C2	−38.5 (2)	C9—C8—C14—O1	−9.2 (3)
Cu1—N2—C2—C1	−39.5 (2)	C9—C8—C14—O2	172.7 (2)
Cu1—N2—C3—C4	58.8 (2)	C9—C10—C11—C12	0.1 (3)
Cu1—N3—C5—C4	−60.6 (2)	C10—C11—C12—C13	0.2 (3)
Cu1—N3—C6—C7	38.7 (2)	C10—C11—C12—C15	179.8 (2)
Cu1—N4—C7—C6	39.9 (2)	C11—C12—C13—C8	−0.3 (3)
N1—C1—C2—N2	52.4 (2)	C11—C12—C15—O3	169.8 (2)
N2—C3—C4—C5	−67.6 (3)	C11—C12—C15—O4	−10.7 (3)
N3—C6—C7—N4	−52.8 (2)	C13—C8—C9—C10	0.2 (3)
C2—N2—C3—C4	−177.47 (18)	C13—C8—C14—O1	169.8 (2)
C3—N2—C2—C1	−167.89 (18)	C13—C8—C14—O2	−8.3 (3)
C3—C4—C5—N3	68.4 (3)	C13—C12—C15—O3	−10.6 (3)
C5—N3—C6—C7	166.35 (18)	C13—C12—C15—O4	168.89 (19)
C6—N3—C5—C4	176.36 (18)	C14—C8—C9—C10	179.2 (2)
C8—C9—C10—C11	−0.3 (3)	C14—C8—C13—C12	−178.94 (19)
C9—C8—C13—C12	0.1 (3)	C15—C12—C13—C8	−179.90 (19)

### Hydrogen-bond geometry (Å, º)

**Table:**

*D*—H···*A*	*D*—H	H···*A*	*D*···*A*	*D*—H···*A*
N1—H1*A*···O2*W*^i^	0.97	2.30	3.143 (2)	145
N1—H1*B*···O3^ii^	0.97	2.07	3.007 (2)	161
N2—H2···O4^iii^	0.98	1.95	2.907 (2)	163
N3—H3···O1	0.98	2.19	3.063 (3)	148
N4—H4*A*···O3*W*^iv^	0.97	2.10	3.042 (2)	163
N4—H4*B*···O3^v^	0.97	2.17	3.054 (2)	151
O1*W*—H1*WA*···O1^iv^	0.87	1.89	2.747 (2)	169
O1*W*—H1*WB*···O4^ii^	0.87	1.89	2.760 (2)	174
O2*W*—H2*WA*···O3^iii^	0.87	2.09	2.930 (2)	161
O2*W*—H2*WB*···O3*W*	0.87	2.00	2.872 (2)	175
O3*W*—H3*WA*···O2^vi^	0.87	2.00	2.823 (2)	157
O3*W*—H3*WB*···O2	0.87	1.85	2.712 (2)	174

Symmetry codes: (i) −*x*, −*y*+1, −*z*+1; (ii) *x*−1, *y*, *z*−1; (iii) *x*−1, *y*, *z*; (iv) *x*, *y*, *z*−1; (v) −*x*+1, −*y*+1, −*z*+1; (vi) −*x*+1, −*y*+1, −*z*+2.
